# Transcriptome analysis of genes involved in defence response in *Polyporus umbellatus* with *Armillaria mellea* infection

**DOI:** 10.1038/srep16075

**Published:** 2015-11-03

**Authors:** Meng-Meng Liu, Yong-Mei Xing, Da-Wei Zhang, Shun-Xing Guo

**Affiliations:** 1 Institute of Medicinal Plant Development, Chinese Academy of Medical Sciences & Peking Union Medical College, Beijing 100193, P. R. China

## Abstract

*Polyporus umbellatus*, a species symbiotic with *Armillaria mellea* and it also exhibits substantial defence response to *Armillaria mellea* infection. There are no genomics resources databases for understanding the molecular mechanism underlying the infection stress of *P. umbellatus*. Therefore, we performed a large-scale transcriptome sequencing of this fungus with *A. mellea* infection using Illumina sequencing technology. The assembly of the clean reads resulted in 120,576 transcripts, including 38,444 unigenes. Additionally, we performed a gene expression profiling analysis upon infection treatment. The results indicated significant differences in the gene expression profiles between the control and the infection group. In total, 10933 genes were identified between the two groups. Based on the differentially expressed genes, a Gene Ontology annotation analysis showed many defence-relevant categories. Meanwhile, the Kyoto Encyclopedia of Genes and Genomes pathway analysis uncovered some important pathways. Furthermore, the expression patterns of 13 putative genes that are involved in defence response resulting from quantitative real-time PCR were consistent with their transcript abundance changes as identified by RNA-seq. The sequenced genes covered a considerable proportion of the *P. umbellatus* transcriptome, and the expression results may be useful to strengthen the knowledge on the defence response of this fungus defend against *Armillaria mellea* invasion.

In nature, organism resistance to exotic species infections is the rule rather than the exception. The defence mechanisms have been extensively studied in plants. For example, to understand the molecular mechanisms of the establishment of the infection of rice with blast fungus, extensive studies have characterized many rice and fungal genes that are involved in plant defence and pathogen attack^1^,^2^,^3^. Meanwhile, a lot of work has been done to explore the molecular mechanism of defence in animals such as *Eriocheir sinensis* and *Portunus trituberculatus*^4^,^5^. But there is little knowledge about the defence system of fungus in the published researches.

*Polyporus umbellatus*, belonging to Polyporaceae in the class of Basidiomycetes has been used as medicine fungus in China for more than 2500 years^6^. The *P. umbellatus* sclerotia can survive in soils for long periods of time, and produce new sclerotia directly from the old ones under appropriate conditions^7^. It is now well known that the growth of *P. umbellatus* sclerotia depends on a symbiotic relationship with the forest pathogenic fungus *Armillaria mellea*^8^, which is a serious root rot disease-causing fungus in the family of Physalacriaceae^9^. In the progress of symbiosis establishment, the rhizomorph of *A. mellea* adheres and invades sclerotia of *P. umbellatus* and the *P. umbellatus* sclerotia launch effective defence responses to fend off *A. mellea* invasion. For example, the cell wall of *P. umbellatus* sclerotia had become irregularly thickened to block the *A. mellea* further invasion and the *P. umbellatus* sclerotia can form a cavity structure in the medullar tissue and isolate *A. mellea* to prevent further intrusion of *A. mellea* hyphae under SEM or TEM observation^10^. Although in previous studies, the preliminary knowledge of interactions between *P. umbellatus* and *A. mellea* has been provided from the morphological characteristics, little information is available on mechanisms of defence response of *P. umbellatus* to *A. mellea* infection up to now.

In recent years, several differential screening techniques (e.g., differential display, subtraction libraries, and differential hybridization) have made it possible to characterize genes that are differentially expressed after certain treatments^11^. Recently, along with the rapid development and cost reduction of next generation sequencing (NGS), sequence-based assays of transcriptomes, namely RNA sequencing (RNA-seq), has been become a comprehensive and accurate tool for gene expression pattern analyses. Compared with the above screening techniques, RNA-Seq is known to have a wider dynamic range, higher technical reproducibility, and provide a better estimate of absolute expression levels^12^. Along with these advantages, RNA-seq has been used to elucidate the response of plants to various environmental stresses, such as cold^13^, salt^14^,^15^ and drought^16^,^17^. However, until now, no genes have been identified, and no molecular research of *Polyporus umbellatus* has been reported, despite the importance of the genus. Furthermore, seldom reports have been found on the response of *P. umbellatus* to *A. mellea* infection.

In this study, in order to identify defence-related genes, potential defence-responsive genes were first identified based on Illumina tag-sequencing, and then, the differentially expressed genes (DEGs) were screened and further validated by qPCR. To our knowledge, this is the first large-scale assessment of Polyporus genomic information. Our results will facilitate understanding of the response of *P. umbellatus* to *A. mellea* infection.

## Results

### Sequencing and *de novo* transcriptome assembly

**Figure 1:**
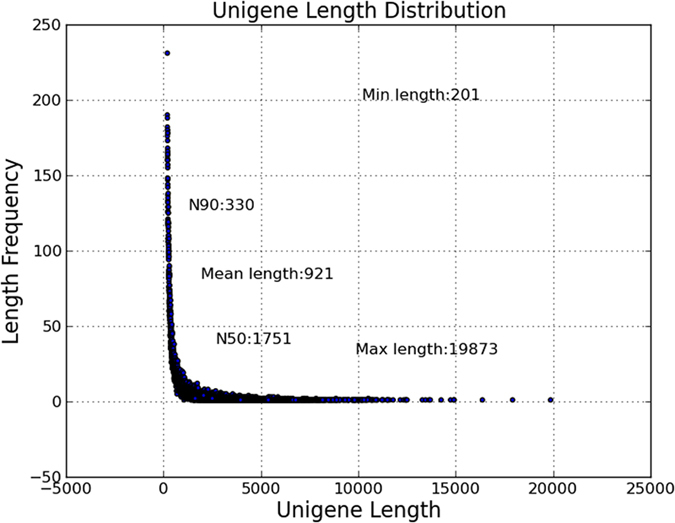
Length distribution of assembled unigenes.

To obtain the *P. umbellatus* sclerotia transcriptome expression profile with *A. mellea* infection, one non-normalized library was constructed using sclerotia tissue with *A. mellea* infection and normal sclerotia tissue as control. Illumina sequencing data from *P. umbellatus* sclerotia was deposited in the NCBI SRA database under accession number SRP058382. In total, 186342740 Illumina PE raw reads were generated (Table 1). After removing adaptor sequences, ambiguous nucleotides and low-quality sequences, there were 177.117254 million clean reads remaining. Assembly of clean reads resulted in 38,444 unigenes in the range of 201–19,873 bp with a N50 length of 1751 bp (Fig. 1).

### Sequence annotation

After eliminating repeated and short-length sequences, 38,444 non-redundant unigenes were subjected to the following 7 public databases (Table 2) for similarity searching. Analyses showed that 24,027 unigenes (62.49%) had significant matches in the Nr database, 2,977 (7.74%) in the Nt database. Our results also showed that 14,214 (36.97%) of non-redundant unigenes demonstrated similarity to the known genes in Swiss-Prot database. In total, there were 25,947 unigenes (67.49%) successfully annotated in at least one of the Nr, Nt, Swiss-Prot, KEGG, GO, COG and Pfam databases, with 1563 unigenes (4.06%) in all seven databases.

**Figure 2:**
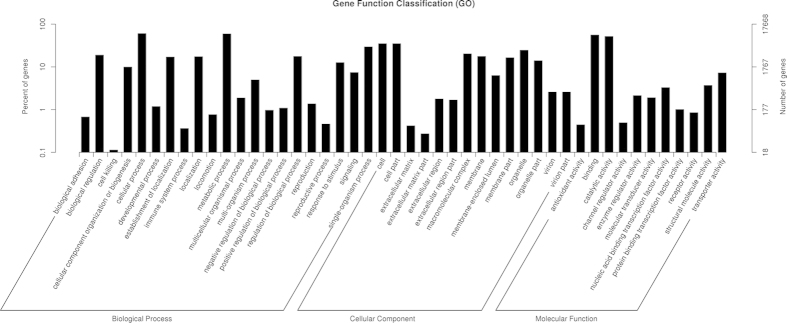
GO categorization of non-redundant unigenes. Each annotated sequence was assigned at least one GO term.

According to Gene Ontology (GO), an international standardized gene functional classification system, 17,688 non-redundant unigenes were classified into three major functional ontologies (biological process, cellular component and molecular function) (Fig. 2). For biological process (B P), dominant subcategories were ‘cellular process’ (10,661) and ‘metabolic process’ (10,466). In the category of cellular component (C C), ‘cell’ (6,188), ‘cell part’ (6,187) and ‘organelle’ (4,329) were highly represented. Among molecular function (M F) terms, ‘binding’ (9,840) and ‘catalytic activity’ (9,126) were most represented, followed by ‘transporter activity’ (1,280). However, within each of the three categories, few genes were assigned to subcategories of ‘cell killing’, ‘extracellular matrix part’ and ‘antioxidant activity’.

**Figure 3:**
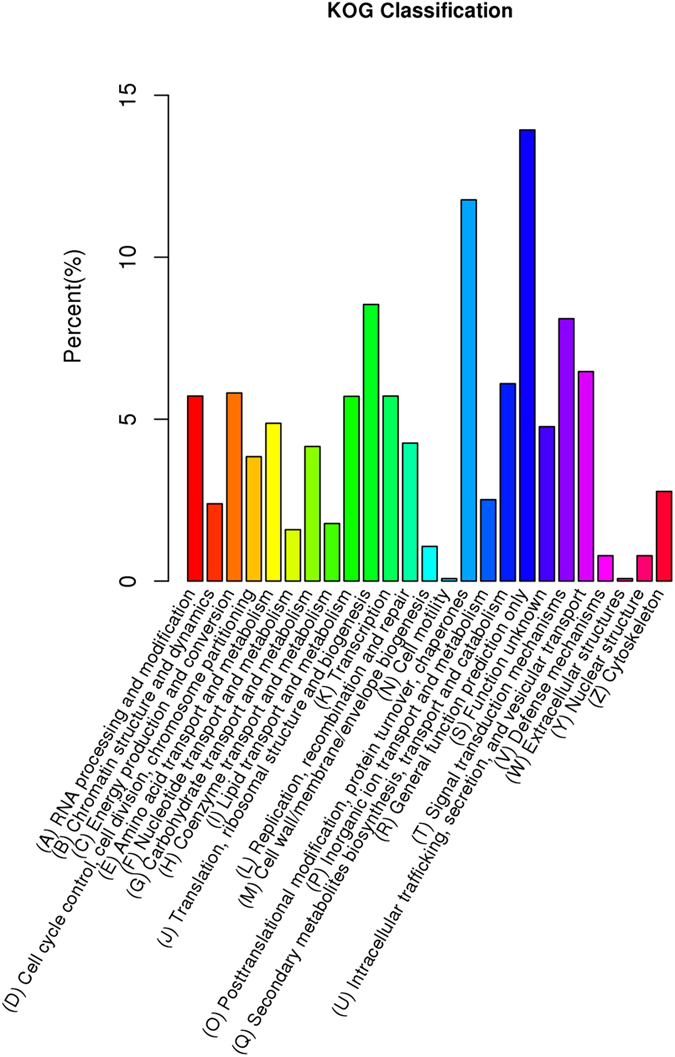
COG annotation of putative proteins.

In addition, all unigenes were subjected to a search against the COG database for functional prediction and classification. 10,466 non-redundant unigenes (Table 2) were subdivided into 25 COG classifications (Fig. 3). Among them, the cluster of ‘general function prediction only’ (1,458) was the largest group, followed by ‘post-translational modification, protein turnover, chaperon’ (1,232), ‘translation’ (894), ‘signal transduction’ (848) and ‘Intracellular trafficking’ (677). Only a few unigenes were assigned to ‘extracellular structures’ (8) and ‘cell motility’ (8).

**Figure 4:**
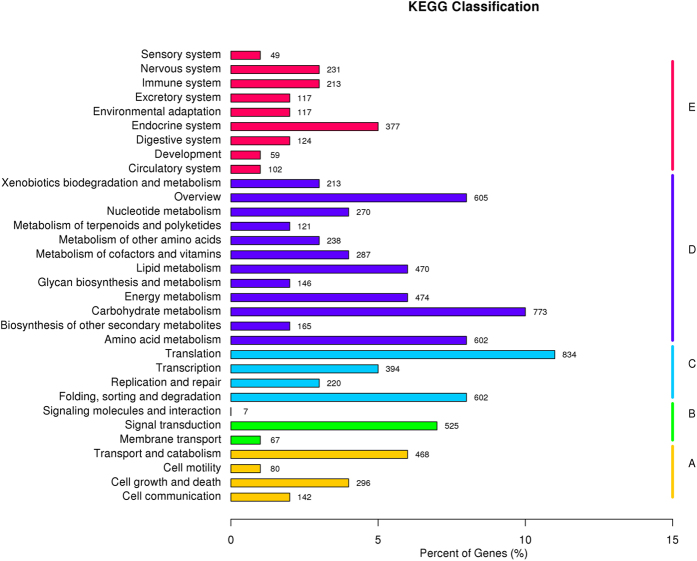
KEGG annotation of putative proteins.

The unigene metabolic pathway analysis was also conducted using the KEGG annotation system. According to KEGG, 7418 unigenes (Table 2) were assigned to 266 pathways (Fig. 4). The pathways involving the largest number of unique transcripts were ‘translation’ (834), followed by ‘carbohydrate metabolism’ (773), whereas ‘signaling molecules and interaction’ (7) was the smallest group.

### Differential expression analysis of assembled *P. umbellatus* transcripts under *A. mellea* infection

**Figure 5:**
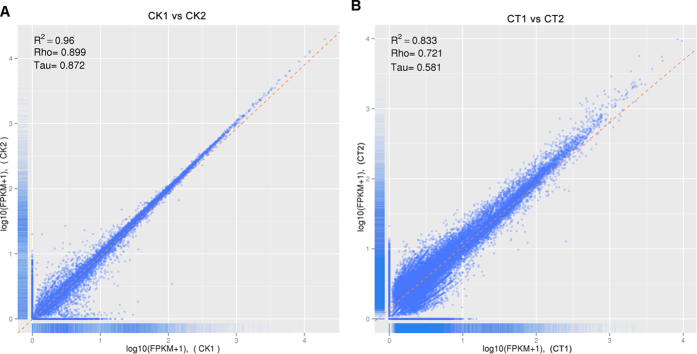
Correlation of RPKM distribution between two biological replicates. Comparisons of estimated RPKM distributions between biological replicates for (**A**) none-invading samples, (**B**) invading with *A. mellea*. Pearson’s correlation coefficients (R^2^) between replicates and statistical significance levels are presented.

To better survey the biological mechanism of defence, it is important to identify the DE genes between two different stages. To increase the accuracies of the measured expression levels for further analyses, data from two biological replicates were merged, and RPKM (Reads Per Kilo-base per Million reads) values were calculated based on the merged dataset. The resulting Pearson’s correlation coefficients (R) were significantly high between the replicates for CT (R = 0.83) and CK (R = 0.96) samples (Fig. 5). Differential expression analysis was firstly performed between the two medullar tissues. DEGs (q-value < 0.005 and |log2 (foldchange)| > 1) were defined as genes that were significantly enriched or depleted in one tissue relative to the other tissue.

**Figure 6:**
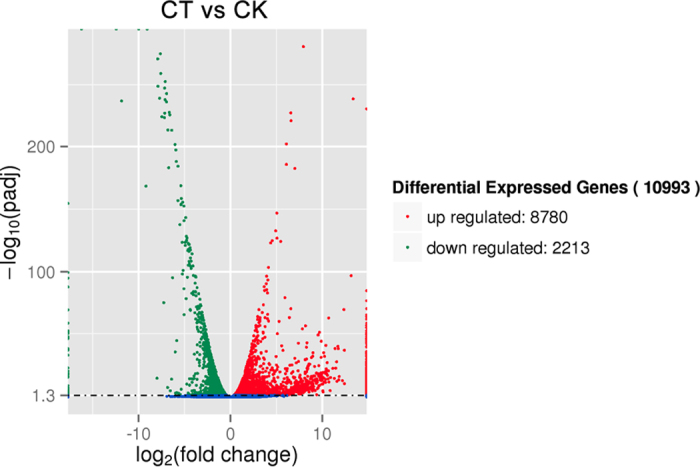
Volcano plot displaying differential expressed genes between CT and CK sample. The y-axis corresponds to the mean expression value of log 10 (p-value), and the x-axis displays the log2 fold change value. The red dots represent the up regulated expressed transcripts (p < 0.05, false discovery rate (FDR) q < 0.05) between CT and CK; the green dots represent the transcripts whose expression down regulated (p < 0.05, FDR q < 0.05).

Then, the DEGs between the CK and CT were analyzed. Of 38,444 unigenes, 10,933 were detected as significantly different by DEseq (Fig. 6). In this study, DEGs with higher expression levels in CT compared with CK were denoted as ‘up-regulated’, while those with lower expression levels in CT were ‘down-regulated’. The expression levels of 8780 of the 10993 genes were up-regulated in the CK while the other 2213 genes showed lower expression in the CK.

### Functional distribution of differentially expressed genes

Differentially expressed genes were considered to be the important cause of defence response. To better survey the biological behavior of defence response, it is necessary to understand the functional distribution of these DE genes in *P. umbellatus* with *A. mellea* infection compared to the *P. umbellatus* without *A. mellea* infection.

GO analysis was conducted for the up-regulated DEGs in CT sample. The GO enrichment of up-regulated DEGs in CT was shown in supplementary Table S1. In the molecular function category, the top three enriched terms were threonine-type endopeptidase activity, threonine-type peptidase activity and sequence-specific DNA binding. In the cell components category, ‘proteasome complex’, ‘membrane part’ and ‘membrane’ were the three dominant enriched terms. For biological process, ‘protein folding’, ‘reductive tricarboxylic acid cycle’, and ‘chromatin assembly or disassembly’ was the mostly highly enriched. The iron transport was influenced, with ‘metal ion transmembrane transporter activity’ and ‘cation-transporting ATPase activity’ also enriched (P-value < 0.05). For the down-regulated DEGs in CK compared to CT sample, ‘response to organic substance’ and ‘cellular component biogenesis’ was the top-two biological process enriched by the down-regulated DEGs. The decline of organic substance and cellular component biogenesis might imply a great inhibition of biosynthetic process in the *P. umbellatus*. Consistent with this ‘biosynthesis’ was the top-three biological process enriched by down-regulated DEGs. The GO enrichment of down-regulated DEGs in CT was shown in supplementary Table S2. In the CK sample, ‘nucleoside monophosphate metabolic process’ and ‘pyrimidine-containing compound biosynthetic process’ were the third and fifth most enriched biological process, respectively. Taken together, proliferation of *P. umbellatus* cells was greatly limited with *A. mellea* infection, which may save much energy.

Biological behaviors of DEGs were complicated; the defence reaction is a coreaction result of many inner-cell metabolic pathways. KEGG pathway enrichment analysis for DEGs also revealed the complicated pathways related to defence response. Arginine and proline metabolism were the top-four enriched pathways (q ≤ 0.05) for the up-regulated DEGs in CT *P. umbellatus* cells, compared with CK, the KEGG pathway enrichment of up-regulated DEGs in the CT was shown in supplementary Table S3. There were 51 genes annotated as involved in this pathway. There were two DEGs annotated as Arginase (ARG), an important immune-related gene - comp31000_c0 and comp30397_c0 -with respectively 43.61- and 2.25-fold increased expression, which may lead to a defence reaction in the *P. umbellatus* cells. For the down-regulated DEGs in CT sample, ribosome were the top-one enriched pathways (q ≤ 0.05), the KEGG pathway enrichment of down-regulated DEGs in the CT was shown in supplementary Table S4. The ribosome is the place where mRNA is translated into protein. There were 11 DEGs annotated as ribosomal protein among the 49 genes annotated as involved in this ribosome pathway and all these 11 DEGs showed at least two fold lower expression in the CT sample. The decelebration of ribosome might imply a great inhibition of biosynthetic process, which is consistent with the biological process of GO analysis as discussed above leading to energy saving.

### Validation of differentially expressed genes using qRT-PCR

**Figure 7:**
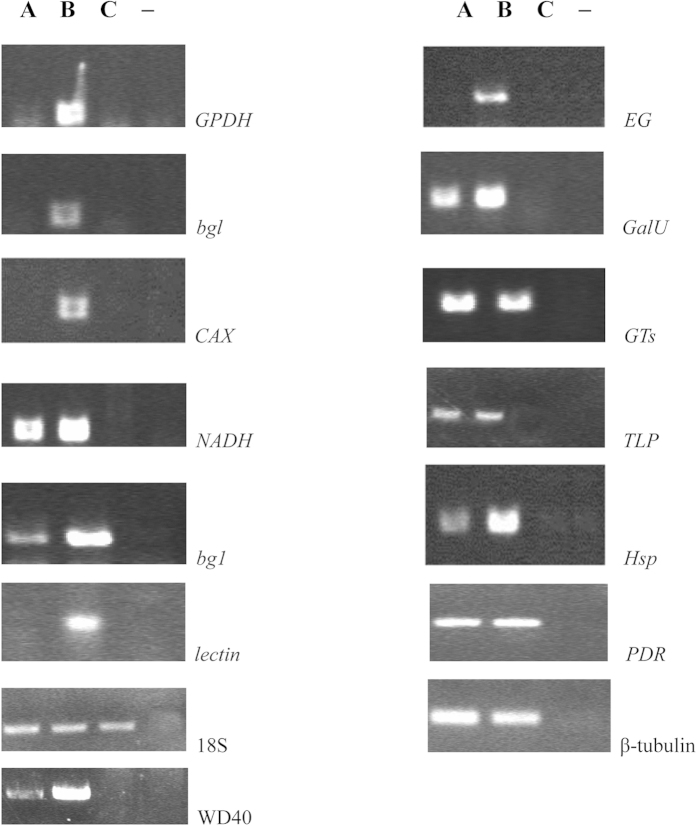
RT-PCR analyses of the *P. umbellatus*. non-infected (**A**) and *P. umbellatus*, infected (**B**) tissues with *A. mellea* and *A. mellea* (**C**). −negative control.

As the cDNA of CT sample was contaminated by the *Armillaria mellea*. Primers designed to the *P. umbellatus* beta-tubulin gene (EU442274) and ribosomal 18S (EU442272), as house-keeping genes, were amplified *P. umbellatus* transcripts in CK, CT cDNA and the genome DNA of *A. mellea*, which was isolated from the hyphae taken from the tissues of the *P. umbellatus* sclerotia^18^ respectively. However, only primers specific for beta-tubulin was not amplified a DNA fragment from the DNA of *A. mellea*, whereas amplicons were obtained for this sample with the ribosomal 18S specific primers which indicated that this beta-tubulin gene was specific in *P. umbellatus* (Fig. 7). For this reason, we only used beta-tubulin primers in the quantitative PCR experiments.

**Figure 8:**
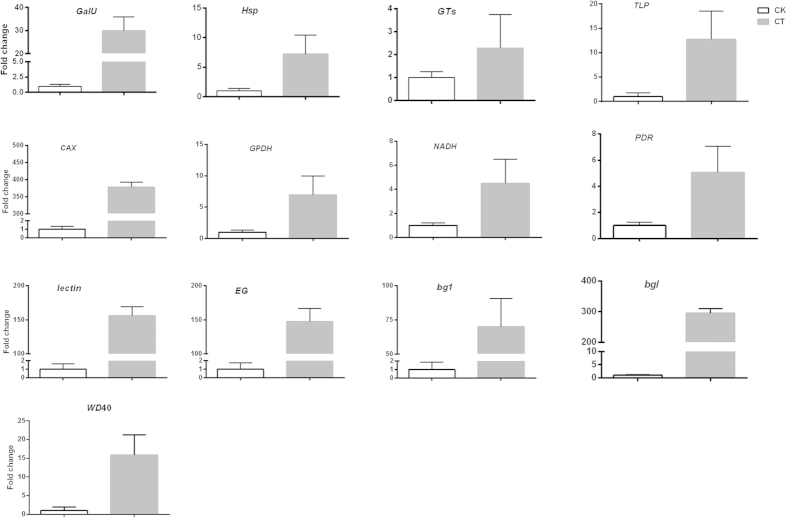
Quantitative real-time PCR (Q-PCR) validations of 12 genes that were differentially expressed between the CK (white bar) and CT (grey bar) samples. For each Q-PCR validation, four technical replications were performed. beta-tubulin gene was used as internal control.

To confirm the reliability of the RNA-Seq data, the transcriptional level of 13 unigenes were examined by real-time quantitative PCR (Fig. 8). All the primers designed to the *P. umbellatus* were shown in supplementary Table S5. All the primers were PCR-amplified in the cDNA of CK, CT samples and genome DNA of *A. mellea* respectively. Only primers were not amplified for the sample of *A. mellea* DNA screened for further real-time quantitative PCR (qRT-PCR) analysis (Fig. 8). Since, new *P. umbellatus* sclerotia materials were used for the RNA extraction, the fold change did not exactly match the number revealed by the DEG analysis for these genes. All the 13 genes exhibited >2 fold higher expression in the *P. umbellatus* in response to *A. mellea* infection. Comp29904_c0 annotated as encoding *lectin* cannot be detected in the CK sample due to no/low expression, just as the result by the Illumina sequencing technology. Taken together, all the unigenes showed consistent expression patterns that were consistent with the RNA-Seq data, indicating that our experimental results were valid.

## Discussion

In this subject, transcriptomes of *P. umbellatus* sclerotia were sequenced using the Illumina platform. In total, about 177 million high-quality reads with 22.16 Gb sequence coverage were obtained; there were 38,444 unigenes (≥200 bp) assembled and 100% were annotated. To the best of our knowledge, this is the first large-scale assessment of *Polyporus* genomic resources. Our results lay the foundation for development of molecular markers, construction of a genetic map and much other genomics research in *Polyporus*. To identify defence-responsive genes, 13 unigenes were selected. The expression of 13 unigenes was significantly up-regulated with *A. mellea* infection.

### Molecular chaperones

The heat-shock proteins (Hsps) as one kind of molecular chaperones, are a ubiquitous feature of cells in which these proteins cope with stress-induced denaturation of other proteins^19^. The roles of Hsps in host-parasite interactions have received considerable attention. From the perspective of the host, Hsps expressed by invading parasites are potent antigens that elicit an immune response^20^,^21^,^22^. In this study, the expression of a small heat shock protein gene was induced by infection. Interestingly, our data agree with the observation that small heat shock protein gene was up-regulated by tobacco etch virus infection in *Arabidopsis*^23^, suggesting that the defence response may induce up-regulation of the small heat shock protein gene.

### Reducing the oxidative damage

The induction of Reactive Oxygen Species (ROS)-related factors suggests that infection stress is accompanied by the production of ROS in organisms, and the induction of enzymes regenerating the reduced forms of antioxidants, and ROS interacting enzymes such as superoxide dismutase, peroxidases and catalases may be involved in the protection of tissues from oxidative damage under infection conditions^24^. The response and dynamics of antioxidant enzyme transcripts, including SOD, POD, PRX, APX and GR *et al.* are commonly used to study plant defence responses^25^. The expression pattern was also observed in this study, in which a GPDH was increased under infection by *A. mellea* in *P. umbellatus*. These results suggest that the antioxidative defence system was greatly activated in *P. umbellatus*. Consistent with the numerous studies that have shown a correlation between the ability to ameliorate ROS and survival under infection conditions, the high induction of ROS-related proteins in infected *P. umbellatus* showed that strong detoxification was critical for its survival.

### Lectins as defence proteins

Lectins are a widespread class of proteins implicated in many essential cellular and molecular recognition processes such as cellular signaling, differentiation, host–pathogen interactions and tissue metastasis^26^. Fungal lectins take part in the defence of fungi since some present toxic activities such as insecticidal, vermicidal or antiviral^27^. For example, the β-trefoil lectins present in the sclerotia^28^ (compacted forms of mycelium) from *S. sclerotiorum* and *R. solani* are toxic towards the aphid *Acyrthosiphon pisum*^29^ and the cotton leafworm *Spodoptera littoralis*^28^, respectively. As expected and verified by qRT-PCR, similar to previous reports, a selected gene encoding lectin from *P. umbellatus* sclerotia was also induced by defence response in this study, suggesting that this *P. umbellatus* lectin have potential utility against *A. mellea* infection.

### Pathogenesis-related proteins (PR-proteins)

Thaumatin-like proteins (TLPs) are a group of PR proteins (PR-5) that are induced in plants in response to infection by plant pathogens, elicitors, stress, and developmental signals^30^. The genes encoding PR-proteins exhibit strong *in vitro* antifungal activity. Several researches have demonstrated that the introduction of a single transgene encoding different antimicrobial proteins including PR-proteins in wheat resulted in enhanced resistance to a wide range of disease resistance^31^. Similarly, similar to the previous reports, a selected TLP from *P. umbellatus* was also induced by defence response in this study, which may suggest that the TLP can inhibit the growth of *A. mellea* for further invasion.

### Secondary metabolism

Uridine triphosphate (UTP)-glucose-1-phosphate uridylyltransferase (GalU) is an enzyme that catalyzes the formation of uridine diphosphate (UDP)-glucose from UTP and glucose-1-phosphate. GalU is involved in virulence in a number of animal-pathogenic bacteria since its product, UDP-glucose, is indispensable for the biosynthesis of virulence factors such as lipopolysaccharide and exopolysaccharide^32^. The up-regulation pattern was also observed in this study, in which a GalU was increased under defence response in *P. umbellatus*. These results suggest that the GalU was a defence-induced gene in *P. umbellatus* and may enhance the virulence to prevent the further invasion of *A. mellea*.

### Cell wall hydrolysis and fusion

The cell wall of orgnism represents an essential protection barrier against infection mechanisms and its integrity is important for the survival of the orgnism^33^. Fungal cell walls are complex structures but typically contain chitin, β-1, β-3- and β-1,6-glucan, mannan and cell wall proteins^34^. In our previous study, we found that *P. umbellatus* sclerotia has formed the cavity structure in the medullar tissue and isolate *A. mellea* to prevent further intrusion of *A. mellea* hyphae^10^. In order to form the cavity, the *P. umbellatus* sclerotia cell wall must first be hydrolyzed and then fused with other cell. The glucanolytic activities of several enzymes have been detected in the cell walls of some kind of fungi, such as *Aspergillus fumigatus, Coccidioides posadasii* and *C. immitis*. Some of these enzymes exhibit both glucanase and transglycosidase activities and, like the glucanases of yeasts, may play some roles in cell wall remodeling during morphogenesis^35^. β-1,3-Glucanases were also found up-regulated in the knob proteome of *Monacrosporiu haptotylum*, suggesting they may be important in fungal morphogenesis^36^. Interestingly, four genes annotated as encoding endoglucanase, beta-glucosidase, β-1,3-glucanase, glycosyltransferase were induced by defence in this research. The large-scale increase of mRNAs associated with glucanase and transglycosidase activities in *P. umbellatus* sclerotia indicated that defence response induces systemic inhibition of cell wall hydrolysis and fusion processes.

### Regulating the fungus efflux

Many cellular processes, including plant responses to pathogens, are regulated by changes in cytosolic Ca^2+^ levels, where Ca^2+^ ions can serve to transduce a particular stimulus or stress that is imposed on a cell to target proteins that guide the cellular response^37^. It is well known that a Ca^2+^ elevation in the cytosol is an important event in plant cell perception of pathogen invasion and the calcium ion is a ubiquitous intracellular second messenger involved in plant defence responses^38^. In this study, a selected calcium/proton exchanger was induced in *P. umbellatus* by *A. mellea* infection (Fig. 8). This result may indicate that the role of calcium/proton exchanger in infection stress is to accumulate the calcium ion for mediating the signaling cascade.

Among the ABC transporters, the pleiotropic drug resistance (PDR) family is particular in that its members are found only in fungi and plants^39^. Several *Saccharomyces cerevisiae* PDR genes, such as PDR5 and SNQ2, have been functionally characterized in detail. They confer resistance to a large number of toxic compounds with no, or little, common structural or functional properties, such as fungicides, herbicides, pesticides, antibiotics, and detergents. PDR transporters are also involved in fungicide resistance of pathogenic fungi such as *Candida albicans*^40^ or *Penicillium digitatum*^41^. There was one unigene annotated as PDR in this study. *Arabidopsis* PDR12 was upregulated in response to oxylipins and the necrotrophic fungus *Botrytis cinerea*^42^,^43^. According to the above discussion, this may facilitate *P. umbellatus* defence response. Additionally, mRNA related to PDR (i.e. multidrug resistance-associated ABC transporter) was highly upregulated in the CT *P. umbellatus*. Thus, more attention needs to be paid to the relationship between PDR with *P. umbellatus* defence tolerance.

### WD40 proteins

WD40 proteins were identified to play a crucial role in diverse protein-protein interactions by acting as scaffolding molecules and thus assisting the proper activity of the proteins^44^. It has been clarified that a subset of WD40 proteins involving in abiotic stress tolerance conducted in *Arabidopsis* and rice. HOS15, a WD40-repeat protein crucial for repression of genes associated with abiotic stress tolerance through histone deacetylation in *Arabidopsis*^45^. SRWD, a novel WD40 protein subfamily was regulated by salt stress in rice^46^. In this study, a unigene encoding for WD40 protein was significantly up-regulated by *A. mellea* infection, a kind of biotic stress, which suggest that the WD40 protein might involve in biotic stress tolerance of *P. umbellatus.* Thus, more attention needs to be paid to the biotic stress tolerance function studies of WD40 in other model plant.

In summary, the expression patterns of 13 putative genes that are involved in defence response were consistent with changes in their transcript abundance, mainly through molecular chaperones, reducing the oxidative damage, PR-proteins, and regulating secondary metabolism, cell wall hydrolysis and fusion. Meanwhile, regulating the fungus efflux plays an important role in defence response in *P. umbellatus*.

## Conclusions

The combination of RNA-seq and DEGs analyses based on Illumina sequencing technology provided comprehensive information on gene expression. The substantially assembled sequences represented a considerable portion of the transcriptome of *P. umbellatus*. Based on the assembled *de novo* transcriptome, 10,933 DEGs were identified between the *P. umbellatus* sclerotial tissues with or without *A. mellea* infection. Kyoto Encyclopedia of Genes and Genomes pathway analysis uncovered that the differentially expressed genes were involved in important pathways, such as ‘metabolic pathways’. In addition, expression patterns of 13 selected DEGs were further validated with qRT-PCR, which reflected significant alteration in major signal pathway and metabolic pathways with the *A. mellea* infection. The information provided here can also further extend the knowledge regarding the molecular basis of the *P. umbellatus* defence reaction at the infection stage with *A. mellea*.

## Methods

### *P. umbellatus* sclerotia collection and preparation

As mentioned above, *P. umbellatus* sclerotia distributes in the nearer soil surface, such as 10–15 cm below the surface. In addition, it was hard to evaluate the infection stage by the infection time because of unlike the rice-pathogen interaction^47^, it took a long time such as 1–2 years for the rhizomorph of *A. mellea* adheres and invades *P. umbellatus* sclerotia^48^. Furthermore, it was difficult to determine the infection stage by the morphological characteristics of sclerotia medullar tissue. Therefore, in this study, we only set up two groups, *P. umbellatus* sclerotia without *A. mellea* infection (CK) and *P. umbellatus* sclerotia with *A. mellea* infection (CT). To increase the accuracies of the project, two biological replicates were included in each transcriptome sample.

**Figure 9:**
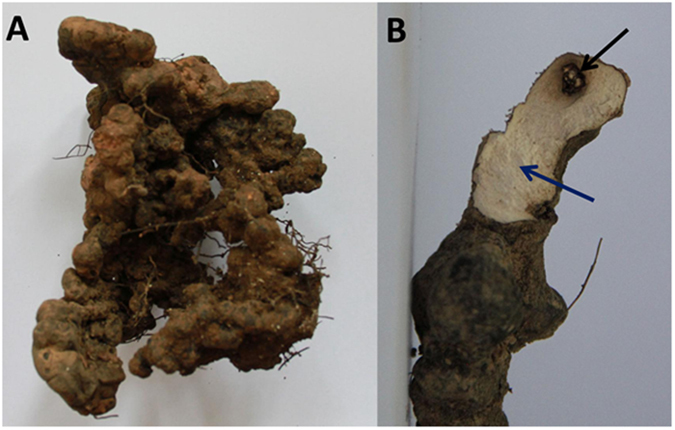
(**A**) The sclerotium of *Polyporus umbellatus* collected from Guxian, Shanxi. The surface of the sclerotium was attached to and penetrated by *A. mellea* rhizomorphs. (**B**) The internal part of the sclerotium. The black region (CT) surrounding the area invaded by rhizomorphs of *A. mellea* (black arrow) and and the none-invading region (CK) was white in color (blue arrow).

*P. umbellatus* sclerotia (Fig. 9A) were collected from Guxian, Shanxi province in July 2014. The sclerotia were surface-disinfected by 75% ethanol for 30s, then cut into half and 100 mg of medullar tissue was removed. The tissue without invaded by the *A. mellea* was administered to the control group (CK), while the part invaded by the *A. mellea* rhizomorphs was treated as test group (CT) (Fig. 9B). Each sample was repeated twice, and all the four samples were frozen in liquid nitrogen immediately after collection and stored at −80 °C prior to RNA extraction.

### RNA extraction, library construction and sequencing

Total RNA was isolated from frozen medullar tissue by using the RNA plant mini kit with column DNAse digestion (Qiagen, Hilden, Germany) following the manufacturer’s instructions. RNA degradation and contamination was detected on 1.2% agarose gels. Then, we measured RNA concentration using Qubit RNA Assay Kit in Qubit 2.0 Flurometer (Life Technologies, Carlsbad, CA, USA). Additionally, RNA integrity was assessed using the RNA Nano 6000 Assay Kit of the Bioanalyzer 2100 system (Agilent Technologies, Santa Clara, CA, USA).

A total amount of 3 μg RNA per sample was used as input material for the RNA preparations. Finally, four samples with RNA integrity number (RIN) values above 8 were used for libraries construction. Sequencing libraries were generated using NEBNext^®^ Ultra™ RNA Library Prep Kit for Illumina^®^ (NEB, USA) following manufacturer’s recommendations and index codes were added to attribute sequences to each sample. Briefly, mRNA was purified from total RNA using poly-T oligo-attached magnetic beads. Fragmentation was carried out using divalent cations under elevated temperature in NEBNext First Strand Synthesis Reaction Buffer. First strand cDNA was synthesized using random hexamer primer and M-MuLV Reverse Transcriptase (NaseH^−^). Subsequently, second strand cDNA synthesis was performed using DNA Polymerase I and RNase H. Remaining overhangs were converted into blunt ends via exonuclease/polymerase activities. After adenylation of 3′ ends of DNA fragments, NEBNext Adaptor with hairpin loop structure was ligated to prepare for hybridization. In order to select cDNA fragments of preferential 150~200 bp in length, the library fragments were purified with AMPure XP system (Beckman Coulter, Beverly, USA). Then 3 μl USER Enzyme (NEB, USA) was used with size-selected, adaptor-ligated cDNA at 37 °C for 15 min followed by 5 min at 95 °C before PCR. Then PCR was performed with Phusion High-Fidelity DNA polymerase, Universal PCR primers and Index (X) Primer. At last, PCR products were purified (AMPure XP system) and library quality was assessed on the Agilent Bioanalyzer 2100 system.

The clustering of the index-coded samples was performed on a cBot Cluster Generation System using TruSeq PE Cluster Kit v3-cBot-HS (Illumia) according to the manufacturer’s instructions. After cluster generation, the library preparations were sequenced on an Illumina Hiseq 2500 platform and 125 bp paired-end reads were generated.

### Sequence reads mapping, assembly and annotation

Raw data (raw reads) of fastq format were firstly processed through in-house Perl scripts. In this step, clean data (clean reads) were obtained by removing reads containing adapter, reads containing ploy-N and low quality reads from raw data. At the same time, Q20, Q30, GC-content and sequence duplication level of the clean data were calculated. All the downstream analyses were based on clean data with high quality.

The *A, mellea* reference genome and gene model annotation files were downloaded from genome website (http://www.icugi.org) directly. The *Armillaria spp.* reads derived from CT group were removed by the mapping of all reads against the *A. mellea* reference genome using Bowtie program (ver. 0.12.8)^49^ with the default parameters. Next, the remaining unmapped non-blast and non- *Armillaria* reads were used to examine the expression profiles of *P. umbellatus*. All the reads were then assembled using Trinity^50^ with min_kmer_cov and were set to 2 and all the other parameters were set to default.

Gene function was annotated based on the following seven databases: Nr (NCBInon-redundantproteinsequences), Nt (NCBI non-redundant nucleotide sequences), Pfam (Protein family), KOG/COG(Clusters of Orthologous Groups of proteins), Swiss-Prot (A manually annotated and reviewed protein sequence database), KO(KEGG Ortholog database) and GO (Gene Ontology), using BLAST with a cutoff E-value of 10^−5^.

### Quantification and differential expression analysis of transcripts

Gene expression levels were estimated by RSEM^51^ for each sample. Clean data were mapped back onto the assembled transcriptome and then the readcount for each gene was obtained from the mapping results.

Differential expression analysis of two groups was performed using the DESeq R package (1.10.1). DESeq^52^ provide statistical routines for determining differential expression in digital gene expression data using a model based on the negative binomial distribution. The resulting P values were adjusted using the Benjamini and Hochberg’s approach for controlling the false discovery rate. Genes with an adjusted P-value < 0.05 found by DESeq were assigned as differentially expressed.

### GO and KEGG enrichment analysis of differentially expressed transcripts

Gene Ontology (GO) enrichment analysis of the differentially expressed genes (DEGs) was implemented by the GOseqR packages based on Wallenius non-central hyper-geometric distribution^53^, which can be adjusted for gene length bias in DEGs.

KEGG^54^ is a database resource for understanding high-level functions and utilities of the biological system, such as the cell, the organism and the ecosystem, from the molecular-level information, especially from large-scale molecular datasets generated by genome sequencing and other high-throughput experimental technologies (http://www.genome.jp/kegg/). We used KOBAS software^55^ to test the statistical enrichment of differential expression genes in KEGG pathways.

### Confirmation of the infection-responsive expression profiles by qRT-PCR

Differentially expressed genes identified by the above described method were validated using quantitative real-time PCR (qPCR). The real-time PCR was performed with the SYBR® Premix ExTaq^TM^ (TaKaRa, Dalian, China) on the ABI 7500 Real-Time PCR System (Applied Biosystems, Foster City, CA, USA). The beta-tubulin gene was used as a reference control. The reaction was performed using the following conditions: denaturation at 95 °C for 30 s, followed by 40 cycles of amplification (95 °C for 5 s, 60 °C for 34 s). Each plate was repeated three times in independent runs for all reference and selected genes. Gene expression was evaluated by the 2^−ΔΔCt^ method^56^.

### Statistical analysis

For each sample, three technical replicates of the qRT-PCR assay were used with a minimum of three biological replicates. Results were expressed as means ± standard deviation (SD) of the number of experiments. A Student’s t-test for the values was performed at P < 0.05.

## Additional Information

**How to cite this article**: Liu, M.-M. *et al.* Transcriptome analysis of genes involved in defence response in *Polyporus umbellatus* with *Armillaria mellea* infection. *Sci. Rep.* **5**, 16075; doi: 10.1038/srep16075 (2015).

## Supplementary Material

Supplementary Information

## Figures and Tables

**Table 1:** Summary of sequences analysis.

Sample	Raw Reads	Clean Reads	Clean Bases	Error (%)	Q20 (%)	Q30 (%)	GC (%)
CT1_1	24750909	23407132	2.93G	0.03	96.91	93.54	55.05
CT1_2	24750909	23407132	2.93G	0.04	93.22	87.58	54.98
CT2_1	21572245	20537549	2.57G	0.03	97.04	93.82	54.76
CT2_2	21572245	20537549	2.57G	0.04	92.54	86.61	54.67
CK1_1	23683593	22616430	2.83G	0.03	96.97	93.60	55.64
CK1_2	23683593	22616430	2.83G	0.04	93.22	87.57	55.53
CK2_1	23164623	21997516	2.75G	0.03	96.87	93.40	55.77
CK2_2	23164623	21997516	2.75G	0.05	92.41	86.21	55.66
Total	186342740	177117254	22.16G				

CT1, CT2: Controlled medullar tissue.

CK1, CK2: Treated medullar tissue.

Q20: The percentage of bases with a Phred value > 20.

Q30: The percentage of bases with a Phred value > 30.

**Table 2:** BLAST analysis of non-redundant unigenes against public databases.

	Number of Unigenes	Percentage (%)
Annotated in NR	24027	62.49
Annotated in NT	2977	7.74
Annotated in KO	7418	19.29
Annotated in SwissProt	14214	36.97
Annotated in Pfam	16270	42.32
Annotated in GO	17668	45.95
Annotated in KOG	10466	27.22
Annotated in all Databases	1563	4.06
Annotated in at least one Database	25947	67.49
Total Unigenes	38444	100
