# Supplementary material for: Transcriptome analysis of genes involved in defence response in Polyporus umbellatus with Armillaria mellea infection
Source: Sci Rep. 2015 Nov 3;5:16075. doi: 10.1038/srep16075 (PMC4630638; doi:10.1038/srep16075)
Supplement: Supplementary Information [file srep16075-s1.doc]

**Transcriptome profiling revealed changes of multiple genes involved in** **defense response in *Polyporus umbellatus* with *Armillaria mellea* infection**

Meng-Meng Liu, Yong-Mei Xing, Da-Wei Zhang, Shun-Xing Guo

Institute of Medicinal Plant Development, Chinese Academy of Medical Sciences & Peking Union Medical College, Beijing 100193, P. R. China

| GO_accession | Description | Term_type | pValue |
| --- | --- | --- | --- |
| GO:0004298 | threonine-type endopeptidase activity | molecular_function | 4.81E-05 |
| GO:0070003 | threonine-type peptidase activity | molecular_function | 4.81E-05 |
| GO:0000502 | proteasome complex | cellular_component | 0.00010897 |
| GO:0006457 | protein folding | biological_process | 0.00035698 |
| GO:0044425 | membrane part | cellular_component | 0.00054522 |
| GO:0043565 | sequence-specific DNA binding | molecular_function | 0.0010503 |
| GO:0019643 | reductive tricarboxylic acid cycle | biological_process | 0.0013155 |
| GO:0006333 | chromatin assembly or disassembly | biological_process | 0.0016692 |
| GO:0008483 | transaminase activity | molecular_function | 0.0019432 |

**Supplementary Table S1.** GO enrichment of up-regulated DEGs in the CT sample

**Supplementary Table S2.** GO enrichment of down-regulated DEGs in CT sample

| GO_accession | Description | Term_type | pValue |
| --- | --- | --- | --- |
| GO:0005198 | structural molecule activity | molecular_function | 0.000488 |
| GO:0005200 | structural constituent of cytoskeleton | molecular_function | 0.0042 |
| GO:0004417 | hydroxyethylthiazole kinase activity | molecular_function | 0.005237 |
| GO:0003735 | structural constituent of ribosome | molecular_function | 0.007076 |
| GO:0010033 | response to organic substance | biological_process | 0.00743 |
| GO:0044085 | cellular component biogenesis | biological_process | 0.008138 |
| GO:0008171 | O-methyltransferase activity | molecular_function | 0.013023 |
| GO:0019028 | viral capsid | cellular_component | 0.014283 |
| GO:0009123 | nucleoside monophosphate metabolic process | biological_process | 0.014901 |

**Supplementary Table S3.** KEGG pathway enrichment of up-regulated DEGs in the CT

| #Term | Sample number | P-Value |
| --- | --- | --- |
| Proteasome | 62 | 1.79E-09 |
| Adherens junction | 23 | 0.002071 |
| Biosynthesis of unsaturated fatty acids | 31 | 0.002959 |
| Arginine and proline metabolism | 51 | 0.003146 |
| beta-Alanine metabolism | 35 | 0.003726 |
| Citrate cycle (TCA cycle) | 43 | 0.004838 |
| MAPK signaling pathway - fly | 7 | 0.007085 |
| TGF-beta signaling pathway | 23 | 0.012976 |
| Ras signaling pathway | 35 | 0.017903 |
| Pyruvate metabolism | 56 | 0.02005 |

**Supplementary Table S4.** KEGG pathway enrichment of down-regulated DEGs in the CT

| #Term | Sample number | P-Value |
| --- | --- | --- |
| Ribosome | 49 | 4.00E-05 |
| Alanine, aspartate and glutamate metabolism | 12 | 0.018151 |
| Pantothenate and CoA biosynthesis | 7 | 0.021351 |
| Glycosylphosphatidylinositol(GPI)-anchor biosynthesis | 6 | 0.037193 |
| Nitrogen metabolism | 6 | 0.048743 |
| Two-component system | 7 | 0.052301 |
| Peroxisome | 15 | 0.054402 |
| Phosphatidylinositol signaling system | 8 | 0.06497 |
| Inositol phosphate metabolism | 8 | 0.091361 |
| Nicotinate and nicotinamide metabolism | 5 | 0.104908 |

**Supplementary Table S5.** Primers for qRT-PCR analysis

| **Accession No.** | **Annotation** | **Primer set** | |
| --- | --- | --- | --- |
|  |  | **Forward primer (5’-3’)** | **Reverse primer (5’-3’)** |
| comp15020_c0 | GPDH | caaaatactggcaaagtggaaa | acctttcatacaccccttcct |
| comp20579_c0 | bgl | ATGACAACTGGAGAGGAGAGTA | CGGCATCTAATTGACGAATGTT |
| comp22715_c0 | CAX | CACCTCTCAGCACTCACTCA | CATCCAACCTACCACAACACAA |
| comp26188_c0 | NADH | CCGAGTCTTCTGGCGTGAT | TCCGAGGCGTAGAGTTCCA |
| comp29904_c0 | lectin | GGTATTCGCACCATTGACAGA | GGAAGACTCATACACGCTCTAC |
| comp30922_c0 | GalU | CTCCTTCGTCCTCGTCACAA | ACATCGTCCGCTGCCTATC |
| comp33865_c0 | Hsp | TCGCTCGCTCACTGAATGG | AAGGTACGCTCGCTCTTGTT |
| Comp34861_c0 | PDR | CCGCACTGTCATTCTCGTATC | CCGTCGTAGTCTCCTGAGTATT |
| comp31131_c0 | GTs | ATAGTCTGGCGAGAGTTGTTGA | AAGGATGCGGTGGAAGGTTAT |
| Comp26507_c2 | bg1 | CAGGTGTTCCGCAATGTCAAGG | GCGAAGACACGAGGTAGGTTCC |
| Comp30459_c0 | EG | GCAGCATCACCAACCTCGTCTT | TCCACCGCCAGTCTCAGTGTT |
| Comp33782_c0 | TLP | TTCTTCGGCGTTCACCTGAGAC | TGCTGTTACCTGGAGTCCCTCT |
| Comp16336_c0 | WD40 | GAAGCATGGACCACACAATG | AAAGTTGCACGGCATTAACC |
| Previously identified | β-tublin | CCTTCCTTGGCAACTCGACA | TCGTCCATACCCTCCTGTGT |
